# Long-term breastfeeding outcomes in very preterm infants: predictors and challenges

**DOI:** 10.3389/fped.2026.1837123

**Published:** 2026-07-22

**Authors:** Ebru Senol, Seyma Karatekin, Alev Bakir Kayı, Leyla Bilgin, Asuman Coban, Gulbin Gokcay, Zeynep Ince

**Affiliations:** 1 Social Pediatrics Doctorate Program, Istanbul University Institute of Health Sciences, Istanbul, Türkiye; 2 Department of Pediatrics, Uskudar University Medical Faculty, Istanbul, Türkiye; 3 Department of Pediatrics, Samsun University Medical Faculty, Samsun, Türkiye; 4 Department of Pediatrics, Division of Social Pediatrics, Istanbul University Medical Faculty, Istanbul, Türkiye; 5 Department of Pediatrics, Division of Neonatology, Istanbul University Medical Faculty, Istanbul, Türkiye

**Keywords:** breastfeeding duration, complementary feeding, exclusive breastfeeding, infant feeding practices, lactation support, very preterm infants

## Abstract

**Background:**

Breastfeeding is considered the gold standard for preterm infant feeding. However, data on breastfeeding continuation beyond the first year of life in very preterm infants are scarce. This study aimed to assess the rates and duration of exclusive and any breastfeeding up to and beyond 24 months of corrected age and to identify maternal, paternal, perinatal, and postdischarge feeding-related factors associated with long-term breastfeeding outcomes.

**Methods:**

This retrospective cohort study included very preterm infants (≤32 weeks’ gestation) born between 2017 and 2019 in a tertiary neonatal intensive care unit. Data were obtained from hospital records, follow-up files, and structured telephone interviews with parents. Feeding methods were evaluated at discharge and at corrected ages of 1, 4, 6, 12, 18, and 24 months, and beyond when needed. Exclusive breastfeeding (EB) duration, total breastfeeding duration, timing of complementary feeding, and reasons for breastfeeding cessation were recorded. Sociodemographic, perinatal, and postdischarge variables were analyzed to determine their association with breastfeeding outcomes.

**Results:**

The rate of EB at discharge was 67.1%, decreasing to 52.1%, 28.8%, and 19.2% at 1, 4, and 6 months, respectively. Breastfeeding rates at 12, 18, and 24 months were 39.7%, 30.1%, and 23.3%. The mean EB duration was 3.5 ± 2.8 months, and total breastfeeding duration was 12.2 ± 10.5 months. Lower parental education levels, younger paternal age, multiple births, partial breastfeeding at discharge, and earlier initiation of formula were significantly associated with shorter EB duration and lower long-term breastfeeding rates. Early initiation of complementary feeding was observed in 50.1% of infants. The most frequent reason for breastfeeding cessation (53.4%) was reported lack or insufficient milk.

**Conclusions:**

This study, which provides the first longitudinal report from Türkiye describing breastfeeding outcomes among very preterm infants up to 24 months and beyond, demonstrated that both breastfeeding rates and durations were lower than recommended. Strengthening breastfeeding support at NICU discharge and maintaining lactation follow-up throughout infancy—particularly for families with lower education levels, younger parents, multiple births, and earlier formula exposure—may improve long-term breastfeeding outcomes in this vulnerable population.

## Background

Breastfeeding is considered the gold standard for preterm infant feeding (1). Human milk, with its complex and adaptive composition of micronutrients, macronutrients, and bioactive and immunological factors, has been shown to protect preterm infants from necrotizing enterocolitis (NEC), late sepsis, bronchopulmonary dysplasia, retinopathy of prematurity (ROP). It also improves developmental outcomes and decreases the risk of metabolic syndrome and insulin resistance in later years of life (1–5).

Preterm infant-mother dyads encounter many obstacles in breastfeeding including initiating lactation, transitioning from tube to oral feeds, and development and maintenance of milk supply (6). Since almost all very preterm babies are hospitalized in neonatal intensive care units (NICU), separation of mother and infant after birth, long-term need for milk expression, low milk supply, and high levels maternal stress become significant breastfeeding challenges (7, 8).

Limited studies from various countries indicate lower breastfeeding rates in preterm infants compared with term infants (9, 10). Mothers of preterm babies often report not being able to breastfeed as long as they desired (11). Lower gestational age, longer NICU stay, lower maternal education, lack of lactation support after discharge, and mixed feeding at discharge are thought to contribute to lower breastfeeding rates among preterm infants (9, 10).

While there are limited number of studies reporting breastfeeding rates among very preterm infants, reports on long-term outcomes are even scarcer (12). Breastfeeding initiation rates are high in many settings, but long-term continuation remains a challenge for very preterm infants. One of the very few studies reporting long term results in very preterm infants, a study from Portugal, has reported a sharp decline in breastfeeding rates after the first year, with continuation rates at 24 months falling to 2%. Multi-regional studies highlight that despite global recommendations, very preterm infants significantly lag behind their term counterparts in achieving breastfeeding milestones in the first year, primarily due to cumulative physiological and social barriers (13).

In Türkiye, breastfeeding is a deeply rooted cultural practice, supported by robust national policies and a high coverage of “Baby-Friendly” hospitals (14). Despite a national median breastfeeding duration of 16.7 months in the general population, preterm infants in our setting remain a high-risk subgroup for early weaning (15). This disparity suggests that favorable national contexts and standard policies may not be sufficient to address the unique lactation challenges faced by families of very preterm infants, highlighting the need for more specialized and prolonged support. Understanding breastfeeding outcomes and associated factors will help provide adequate lactation support and improve breastfeeding outcomes locally and globally. This study aimed to investigate breastfeeding rates among very preterm infants at discharge and at corrected ages of 1, 4, 6, 12, 18 and 24 months; to determine the durations of exclusive and total breastfeeding; and to identify factors associated with breastfeeding discontinuation.

## Methods

### Design and setting

This retrospective cohort study was conducted in the Institute's NICU and high-risk infant clinic to assess breastfeeding rates within their real-world context. The study was approved by the Clinical Research Ethics Board (No:2021/581, 02 April 2021). Written informed consent was obtained from the parents of all infants.

Breastfeeding is highly prevalent in our country, with approximately 98% of the births occurring in baby-friendly hospitals equipped with adequate lactation support (14). According to the 2018 national survey, 98% of infants are breastfed, with a median duration of 16.7 months; 34% continue up to 2 years (16).

Our hospital was designated baby friendly in 2006 and our tertiary NICU was designated as baby friendly in 2019. Within the unit, care and feeding practices adhere to baby-friendly NICU principles, and the unit undergoes regular inspections by the Ministry of Health every five years (16). All newborns are primarily fed with their mother's milk; formula milk is used only when breast milk is unavailable, as there are no milk banks. Following discharge, all preterm infants are scheduled for follow-up in the high-risk infant outpatient clinic run by the NICU team.

The sample comprised preterm infants born at or less than 32 weeks of gestation in the years 2017, 2018 and 2019, who were treated in the NICU and were successfully discharged. Exclusion criteria included severe maternal physical or mental illness, contraindications for breastfeeding at birth, major congenital anomalies, language and communication barriers with the family, death of the infant or mother within two years following birth, and the presence of short bowel syndrome in the infant.

### Data collection and variable definitions

Data were collected from neonatal intensive care unit (NICU) medical records and high-risk infant outpatient clinic records. Missing or incomplete information was gathered via structured telephone interviews conducted by the leading researcher.

Data regarding the study variables were systematically extracted and analyzed to provide a comprehensive profile of the cohort. Maternal and paternal characteristics included education levels, ages, occupation, family income, and family structure, while maternal health and pregnancy information encompassed pre-existing chronic diseases, pregnancy-related complications, multiple gestation, and previous breastfeeding experience. Neonatal data consisted of birth weight, gestational age (estimated by the obstetrics team based on ultrasonography and last menstrual period), gender, and the type of delivery. The clinical course was documented through variables such as the duration of NICU stay, the time required to achieve first total enteral and oral feedings, the time required to achieve full enteral and oral feedings, and the mode of feeding at the time of discharge. Also diagnoses including respiratory distress syndrome (RDS), necrotizing enterocolitis (NEC), patent ductus arteriosus (PDA), bronchopulmonary dysplasia (BPD), and retinopathy of prematurity (ROP) were noted. Additionally, postdischarge feeding data were collected to record breastfeeding status at corrected ages of 1, 4, 6, 12, 18, and 24 months (and beyond when available); duration of exclusive breastfeeding; total breastfeeding duration; reason for weaning; whether the mother breastfed as much as planned or desired; timing of complementary feeding; and formula use.

In our study, all infants were followed longitudinally until the complete cessation of breastfeeding, regardless of the 24-month milestone. For infants whose breastfeeding continued past 24 months, follow-up remained active through phone calls or clinical visits until the exact date of weaning was recorded. Therefore, the duration data used in our analysis represent the actual completed breastfeeding periods rather than right-censored observations at a fixed time point.

Feeding definitions: Feeding status for infants younger than six months of corrected age was categorized as exclusive breastfeeding (EB), partial breastfeeding (PB), or no breastfeeding. Exclusive breastfeeding was defined as feeding with breast milk only, without any additional foods or liquids (including water), except prescribed medications or human-milk fortifiers. Partial breastfeeding referred to any breastfeeding in combination with other foods or liquids, including water. For infants older than six months of corrected age, breastfeeding status was categorized as breastfeeding or no breastfeeding. Early complementary feeding is defined as the introduction of solid or semi-solid foods before the 6th month of corrected age.

### Data analysis

Statistical analysis was performed using Statistical Package for the Social Science (SPSS, version 28.0; IBM Corp., Armonk, NY, USA).

Descriptive statistics were presented as frequencies and percentages for categorical variables and as mean ± standard deviation (SD) or median (min–max) for continuous variables, as appropriate.

Comparisons between categorical variables—including breastfeeding rates, demographic characteristics, and NICU diagnoses—were conducted using the Chi-square test or Fisher's exact test. Normality of distribution for continuous variables such as demographic data, gestational age, weight, and breastfeeding durations was assessed using the Shapiro–Wilk test. For group comparisons, the independent-samples *t*-test was applied to normally distributed data and the Mann–Whitney *U* test to non-normally distributed data.

When comparing more than two groups, one-way analysis of variance (ANOVA) was used for normally distributed variables and the Kruskal–Wallis test for non-normally distributed variables. Correlations between two continuous variables were analyzed using Pearson's correlation for normally distributed data and Spearman's rank correlation for non-normally distributed data. A two-tailed *p*-value < 0.05 was considered statistically significant, and results were reported with a 95% confidence interval. Odds Ratios (ORs) with 95% Confidence Intervals (CIs) were calculated using univariate analyses to assess the strength of associations between independent variables and breastfeeding outcomes, and statistically significant results were highlighted.

## Results

Between 2017 and 2019, a total of 92 infants who met the study criteria were treated in the NICU and successfully discharged. Of these, 14 infants were excluded from the study in the follow-up period: 5 infants died before 1 year of age, 1 infant's mother died within the first year, 1 family declined participation, and 7 infants who were not followed up in our high-risk infant clinic could not be reached. The remaining 78 infants were included in the study.

Of 78 infants included, 46.2% (*n* = 36) were girls. The mean gestational age was 29.6 ± 1.8 weeks and the mean birth weight was 1224 ± 432 g. 28.2% (*n* = 22) of the infants were conceived by *in vitro* fertilization. 53.8% (*n* = 42) were singletons, while 42.3% (*n* = 33) were twins and 3.8% (*n* = 3) were triplets. 94.9% (*n* = 74) were delivered by cesarean section. Group characteristics are summarized in Table 1.

**Table 1:** Group characteristics.

Variables	*n* (%) or Median (min-max)
Gestational age (weeks)	
Median (min-max)	30.2 (23.4–32.0)
Mean (SD)	29.6 (1.8)
Birth weight (g)	
Median (min-max)	1,147 (460–2,280)
Mean (SD)	1,224 (432)
Maternal age (years)	
Median (min-max)	32 (20–49)
Mean (SD)	33.0 (6.2)
Paternal age (years)	
Median (min-max)	35 (24–49)
Mean (SD)	35.5 (5.7)
Maternal education level	
Elementary	26 (33.3%)
Middle/high school and above	52 (66.7%)
Paternal education level	
Elementary	22 (28.2%)
Middle/high school and above	56 (71.8%)
RDS diagnosis	40 (51.3%)
Ventilation duration (days)	
Median (min-max)	2.0 (0–91)
Mean (SD)	10.6 (18.8)
NEC	
None/Stage 1	72 (92.3%)
Stage 2/3	6 (7.7%)
PDA diagnosis	27 (34.6%)
BPD	
None/mild	57 (73.1%)
Moderate/severe	21 (26.9%)
Length of NICU stay (days)	
Median (min-max)	43.4 (15–159)
Mean (SD)	50.1 (26.3)

RDS, respiratory distress syndrome; NEC, necrotising enterocolitis; PDA, patent ductus arteriosus; BPD, bronchopulmonary dysplasia; NICU, neonatal intensive care unit.

Feeding characteristics of the study group are summarized in Table 2. At discharge, 93.6% (*n* = 73) were breastfeeding. Five infants who were not receiving any breast milk at the time of NICU discharge were excluded from the longitudinal follow-up and duration analyses. This exclusion was pre-specified to ensure that the cohort represent a population capable of achieving breastfeeding initiation, thereby allowing for a focused analysis of factors associated with the maintenance and cessation of breastfeeding over time.

**Table 2:** Feeding characteristics of the study group in NICU.

Feeding Parameters	*n* (%) or Median (min-max)
Prior breastfeeding experience	
No	46 (58.9%)
Yes	32 (41.1%)
First feeding time (days)	
Median (min-max)	2 (1–17)
Mean (SD)	2.6 (2.5)
First feeding content	
Mother's own milk	67 (85.9%)
Formula	11 (14.1%)
Full enteral feeding time (days)	
Median (min-max)	11 (4–44)
Mean (SD)	12.8 (8.5)
Full oral feeding time (days)	
Median (min-max)	39 (1–156)
Mean (SD)	40.5 (22.1)
Length of stay (days)	
Median (min-max)	43 (15–159)
Mean (SD)	50.1 (26.3)
Discharge weight (g)	
Median (min-max)	2,060 (1,400–3,500)
Mean (SD)	2,071 (426)
Breastfeeding status at discharge	
Exclusive breastfeeding	49 (62.8%)
Partial breastfeeding	24 (30.8%)
No breastfeeding	5 (6.4%)

Breastfeeding rates of our group at discharge, corrected 1, 4, 6, 12, 18 and 24 months are presented in Table 3.

**Table 3:** Breastfeeding rates of the group.

Breastfeeding status	Discharge	1 month	4 months	6 months
Exclusive breastfeeding	49 (67.1%)	38 (52.1%)	21 (28.8%)	14 (19.2%)
Partial breastfeeding	24 (32.8%)	31 (42.4%)	36 (49.3%)	31 (42.5%)
No breastfeeding		4 (5.5%)	16 (21.9%)	28 (38.3%)
Total	73 (100%)	73 (100%)	73 (100%)	73 (100%)

Breastfeeding status	6 months	12 months	18 months	24 months
Breastfeeding	45 (61.6%)	29 (39.7%)	22 (30.1%)	17 (23.3%)
No breastfeeding	28 (38.4%)	44 (60.3%)	51 (69.9%)	56 (76.7%)
Total	73 (100%)	73 (100%)	73 (100%)	73 (100%)

The relationship between all collected variables and breastfeeding status was analyzed for discharge and each corrected age point. All reported Odds Ratios (ORs) are unadjusted and derived from 2 × 2 comparisons, not from multivariable logistic regression models. Only statistically significant factors are described below. Parameters associated with breastfeeding at 1, 4 and 6 months are shown in Table 4 and those associated with 12, 18 and 24 months in Table 5.

**Table 4:** Parameters associated with breastfeeding status at 1, 4 and 6 months.

Variables		Breastfeeding status at 1 month			Breastfeeding status at 4 months				Breastfeeding status at 6 months			
		EB (*n* = 38)	Not EB (*n* = 35)	*p*	EB (*n* = 21)	PB (*n* = 36)	NB (*n* = 16)	*p*	EB (*n* = 14)	PB (*n* = 31)	NB (*n* = 28)	*p*
Maternal age (years)		33.8 (5.8)	31.7 (6.6)	0.240^a^	34.4 (6.0)	32.3 (6.7)	31.0 (4.7)	0.203^d^	34.2 (6.5)	33.2 (5.8)	31.1 (6.4)	0.210^d^
Paternal age (years)		36.7 (5.3)	34.1 (6.1)	0.078^a^	38.3 (5.6)	35.0 (5.9)	32.5 (4.3)	**0** **.** **010** ^d^	38.0 (5.7)	36.4 (5.4)	33.0 (5.5)	**0** **.** **014** ^d^
Maternal education level	Elementary	9 (23.7%)	17 (48.6%)	**0** **.** **048** ^b^	5 (23.8%)	17 (47.2%)	4 (25%)	0.124^b^	3 (21.4%)	13 (41.9%)	10 (35.7%)	0.394^b^
	Higher	29 (76.3%)	18 (51.4%)		16 (76.2%)	19 (52.8%)	12 (75%)		11 (78.6%)	18 (58.1%)	18 (64.3%)	
Paternal education level	Elementary	6 (15.8%)	16 (45.7%)	**0** **.** **011** ^b^	4 (19%)	13 (36.1%)	5 (31.3%)	0.379^b^	2 (14.3%)	11 (35.5%)	9 (32.1%)	0.304^b^
	Higher	32 (84.2%)	19 (54.3%)		17 (81%)	23 (63.9%)	11 (68.8%)		12 (85.7%)	20 (64.5%)	19 (67.9%)	
IVF status	No	28 (73.7%)	25 (71.4%)	1.0^a^	15 (71.4%)	29 (80.6%)	9 (56.3%)	0.202^b^	8 (57.1%)	27 (87.1%)	18 (64.3%)	**0** **.** **044** ^b^
	Yes	10 (26.3%)	10 (28.6%)		6 (28.6%)	7 (19.4%)	7 (43.8%)		6 (42.9%)	4 (12.9%)	10 (35.7%)	
Gestational age (weeks)		29.5 (1.8)	29.8 (1.8)	0.417^c^	29.2 (1.9)	29.9 (1.5)	29.6 (2.2)	0.392^d^	28.9 (2.0)	29.5 (1.5)	30.1 (1.9)	**0** **.** **044** ^d^
Singleton	Yes	26 (68.4%)	14 (40%)	**0** **.** **028** ^a^	15 (71.4%)	19 (52.8%)	6 (37.5%)	0.114^b^	10 (71.4%)	19 (61.3%)	11 (39.3%)	0.090^b^
	No	12 (31.6%)	21 (60%)		6 (28.6%)	17 (47.2%)	10 (62.5%)		4 (28.6%)	12 (38.7%)	17 (60.7%)	
Prior BF experience	No	16 (42.1%)	14 (40%)	1.0^b^	12 (57.1%)	17 (47.2%)	14 (87.5%)	**0** **.** **024** ^b^	9 (64.3%)	13 (41.9%)	21 (75%)	**0** **.** **033** ^b^
	Yes	22 (57.9%)	21 (60%)		9 (42.9%)	19 (52.8%)	2 (12.5%)		5 (35.7%)	18 (58.1%)	7 (25%)	
Age at first full enteral feeding (days)		14.1 (8.4)	11.2 (8.6)	**0** **.** **032** ^a^	14.2 (9.2)	12.6 (9.0)	11.0 (6.5)	0.570^d^	14.7 (8.8)	14.8 (9.8)	9.3 (5.6)	**0** **.** **016** ^d^
Age at first full oral feeding (days)		44.6 (26.5)	35.6 (17.2)	0.112^a^	52.0 (31.1)	36.1 (14.4)	33.4 (20.7)	**0** **.** **049** ^d^	62.2 (37.4)	40.2 (14.2)	30.8 (16.7)	**0** **.** **001** ^d^
Age at first formula feed (months) (*n* = 59)		N/A	N/A		7.4 (2.9)	0.73 (2.8)	−0.2 (2.3)	<**0.001**^d^	10.8 (3.7)	1.7 (3.0)	−0.3 (2.2)	**<0** **.** **001** ^d^

*Bold values indicate statistically significant associations (*p* < 0.05).

Continuous variables are presented as Mean (SD), and categorical variables are presented as *n* (%). IVF, *in vitro* fertilization; BF, breastfeeding; EB, exclusive breastfeeding; PB, partial breastfeeding; NB, no breastfeeding.

a Independent samples *t* test.

b Chi-square test.

c Mann–Whitney *U* test.

d Independent samples Kruskal–Wallis test.

**Table 5:** Parameters associated with breastfeeding status at 12, 18 and 24 months.

Variables		Breastfeeding status at 12 months			Breastfeeding status at 18 months			Breastfeeding status at 24 months		
		Breastfeeding (*n* = 29)	No breastfeeding (*n* = 44)	*p*	Breastfeeding (*n* = 22)	No breastfeeding (*n* = 51)	*p*	Breastfeeding (*n* = 22)	No breastfeeding (*n* = 51)	*p*
Prior breastfeeding experience	No	14 (48.3%)	29 (65.9%)	0.209^a^	8 (36.4%)	35 (68.6%)	**0** **.** **021** ^a^	8 (47.1%)	21 (37.5%)	0.394^a^
	Yes	15 (51.7%)	15 (34.1%)		14 (63.6%)	16 (31.4%)		9 (52.9%)	35 (62.5%)	
Breastfeeding at discharge	Exclusive	26 (89.7%)	23 (52.3%)	**0** **.** **002** ^a^	19 (86.4%)	30 (58.8%)	**0** **.** **043** ^a^	14 (82.4%)	35 (62.5%)	0.218^a^
	Partial	3 (10.3%)	21 (47.7%)		3 (13.6%)	21 (41.2%)		3 (17.6%)	21 (37.5%)	
Age at first formula feed (months) (*n* = 59)		4.3 (5.4)	0.7 (3.2)	**0** **.** **007** ^b^	1.4 (2.5)	1.7 (4.4)	0.647^b^	1.6 (2.5)	1.6 (4.4)	0.603^b^
Exclusive breastfeeding duration (months)		4.1 (3.0)	0.4 (2.5)	**<0** **.** **001** ^b^	3.6 (2.9)	1.1 (3.1)	**0** **.** **002** ^b^	3.3 (2.7)	1.4 (3.3)	**0** **.** **030** ^b^

*Bold values indicate statistically significant associations (*p* < 0.05).

Continuous variables are presented as Mean (SD), and categorical variables are presented as *n* (%).

a Chi-square test.

b Mann–Whitney *U* test.

Regarding early outcomes, exclusive breastfeeding at 1 month was significantly associated with single births, with these infants being more than three times as likely to be exclusively breastfed at 1 month compared to multiples (OR: 3.25, 95% CI: 1.24–8.50, *p* = 0.028). A similar association was observed regarding parental education; infants of fathers with only elementary education had higher odds of not achieving exclusive breastfeeding (OR: 4.49, 95% CI: 1.49–13.51, *p* = 0.011) compared to those with higher education. Maternal education level followed a comparable pattern (OR: 3.02, 95% CI: 1.03–8.86, *p* = 0.048).

In terms of long-term outcomes, exclusive breastfeeding at discharge showed a strong positive association with continued breastfeeding. Infants in the exclusive group at discharge demonstrated nearly eight-fold higher odds of breastfeeding at 12 months (OR: 7.91, 95% CI: 2.09–30.00, *p* = 0.002) and four-fold higher odds at 18 months (OR: 4.43, 95% CI: 1.13–17.3, *p* = 0.043) than those who received partial breastfeeding. Furthermore, a significant link was found between the timing of formula introduction and long-term outcomes; infants still breastfed at 12 months had a significantly later introduction of formula, with a mean difference of 3.6 months (95% CI: 1.1–6.1, *p* = 0.007).

Exclusive and total breastfeeding durations of the group are presented in Table 6.

**Table 6:** Exclusive breastfeeding and breastfeeding durations of the group.

Breastfeeding duration parameters	Median (min-max) and Mean (SD)
Exclusive Breastfeeding duration of infants who were exclusively breastfed at discharge (chronological) (*n* = 49)	
Median (min-max)	6.0 months (0.7–14.7 months)
Mean (SD)	6.1 (2.9) months
Exclusive breastfeeding duration of infants who were exclusively breastfed at discharge (corrected age) (*n* = 49)	
Median (min-max)	3.5 months (36 weeks–12 months)
Mean (SD)	3.5 (2.8) months
Breastfeeding duration (chronological) (*n* = 73)	
Median (min-max)	12 months (1.2–48 months)
Mean (SD)	14.8 (10.6) months
Breastfeeding duration (corrected age) (*n* = 73)	
Median (min-max)	9 months (36 weeks–44.5 months)
Mean (SD)	12.2 (10.5) months

Among infants from multiple pregnancies, the mean total breastfeeding duration was 9.8 ± 10.6 months which was statistically shorter than singletons (14.3 ± 10.1 months *p*:0.007). Infants of mothers with prior breastfeeding experience had a mean breastfeeding duration of 16.0 ± 10.9 months, compared with 9.6 ± 9.4 months among infants of mothers without prior experience (*p*:0.005).

Breastfeeding status at discharge also had a statistically significant association with total duration: infants EB at discharge had a mean duration of 14.9 ± 10.6 months, whereas those partially breastfeeding at discharge had 6.8 ± 8.0 months (*p*:0.001).

It was observed that 80.8% (*n* = 59) of the preterm group were introduced to formula feeding at some point. Among those who received formula (*n* = 59), total breastfeeding duration showed a moderate positive correlation with the age of formula introduction (*p* < 0.001 *r*:0.52); the later the formula feeding was introduced, the longer the total breastfeeding duration.

The most common reason for cessation of breastfeeding was “milk supply related problems” (53.4%, *n* = 39) including insufficient milk supply or infants stopping sucking. This was followed by “perceived sufficient duration of breastfeeding” (26%, *n* = 19), “maternal medical problems” (12.3%, *n* = 9), “concern that infant was not receiving enough milk” (4.1%, *n* = 3), “new pregnancy” (2.7%, *n* = 2) and “to encourage solid feeding” (1.4%, *n* = 1).

Of 72 mothers who answered the question whether they had breastfed as long as desired, 61.1% (*n* = 44) answered “no”.

The mean corrected age for introduction of complementary feeding was 5.0 ± 1.6 months (median:5.5 range: 2–12 months). In our cohort, 32% (*n* = 23) of infants started complementary feeding before 4 months, 19% (*n* = 14) at 4–5 months and 49% (*n* = 36) were introduced at 6 months or later. Among infants introduced before 6 months, 24 (65%) started at 6 months of chronological age rather than 6 months of corrected age.

## Discussion

Studies on the breastfeeding status of preterm infants are limited in the literature and vary in gestational age groups, follow-up durations, breastfeeding status definitions, and the use of chronological or corrected age. This study contributes to the literature as one of the first to evaluate long-term breastfeeding outcomes with follow-up extending over two years.

Exclusive breastfeeding rates at discharge reported in the literature range from 34% to 84% for similar gestational age groups (17–22). A Swedish study reported EB rates of 38% at discharge among infants <28 weeks and 34% for infants 28–31 weeks, whereas a French study found 38% among infants <32 weeks (17, 18). In contrast, a larger Danish study involving 478 preterm infants reported a 60% EB rate at discharge (22). An Australian study which included 49 infants born at 24–33 weeks reported an EB rate of 84% at discharge (23). Our study's EB rate of 62.8% falls within the upper range of these findings, possibly reflecting the strong cultural support for breastfeeding and the baby-friendly NICU practices in our setting.

The World Health Organization recommends exclusive breastfeeding for the first six months and continuation of breastfeeding until two years of age or beyond for both term and preterm infants. However, consistent with prior reports, our results show that very preterm infants have lower breastfeeding rates than term infants. A study of term infants in Türkiye found EB rates of 68.7% at 4 months and 35.1% at 6 months, whereas in our cohort, rates were 28.8% and 19.2%, respectively (24). Studies from China and Australia have reported similar patterns, with preterm EB rates at 6 months remaining below those of term infants (6, 9, 10).

Our study also found total breastfeeding rates of 39.7%, 30.1%, and 23.3% at 12, 18, and 24 months, respectively—lower than the national 2-year rate of 34% reported in the Turkish population survey. These findings highlight the ongoing challenge of maintaining long-term breastfeeding in preterm populations (15).

When compared with studies of similar cohorts (Table 7), our group demonstrated relatively higher EB rates at early follow-up points. This may be explained by cultural acceptance of breastfeeding, strong NICU lactation support, and structured follow-up in our high-risk infant clinic. However, even these rates remain below the WHO's global nutrition target of a 50% EB rate at 6 months (23).

**Table 7:** Comparison of breastfeeding rates with similar groups (12, 17, 25).

Countries	Exclusive breastfeeding rates		
	1 month	4 months	6 months
Sweden (*n* = 132)^a^	44%^c^	26%	4%
Denmark (*n* = 317)^a^	35–41%	12–17%	2%
Türkiye (*n* = 73)^a^	52.1%	28.8%	19.2%

	Breastfeeding rates		
	6 months	12 months	24 months
Sweden (*n* = 292)^a^	N/A	21%	N/A
Portugal (*n* = 466)^b^	N/A	10.2%	2%
Denmark (*n* = 317)^a^	28–30%	3–7%	N/A
Türkiye (*n* = 73)^a^	61.6%	39.7%	23.3%

a Data reported in corrected age.

b Data reported in chronological age.

c 2nd month data.

N/A, not available.

Regarding long-term outcomes, our observed breastfeeding rate of 23.3% at 24 months corrected age is notably higher than the few existing international longitudinal reports for very preterm infants. For instance, a prominent Portuguese cohort reported a sharp decline to only 2% at the same milestone (12). This disparity suggests that despite the physiological and social barriers inherent to prematurity, the high cultural value placed on breastfeeding in Türkiye, combined with the “Baby-Friendly” hospital environment, may foster greater resilience in long-term continuation. However, variations in inclusion criteria, follow-up completeness, and breastfeeding definitions may also be contributing to differences between cohorts.

Furthermore, when compared to one of the largest European multi-center cohorts, which documented a significant attrition in breastfeeding rates only within the first six months of life (34% in the European cohort vs. 61.6% in our study), our findings indicate a more sustained trajectory (13). While that large-scale study focused on early-stage discontinuation, our data extends the evidence base significantly by providing a 24-month perspective. This highlights not only the effectiveness of localized follow-up and cultural support in our setting but also addresses a critical gap in the literature regarding the long-term maintenance of breastfeeding in very preterm populations.

At the first month, sociodemographic factors showed a significant association with early lactation success**.** Higher parental education levels were significantly associated with exclusive breastfeeding, with infants of fathers with only elementary education showing a 4.49-fold higher likelihood of not achieving EB at 1 month. Similarly, lower maternal education associated with a 3-fold increase in the likelihood of non-exclusive breastfeeding. Additionally, singleton infants were more than three times as likely to be exclusively breastfed at 1 month compared to multiples. Higher paternal age was associated with exclusive and partial breastfeeding at 4 and 6 months. A large European study involving very preterm infants reported that younger and less educated mothers had lower breastfeeding rates at 6 months (13). Comparable results were observed in Israel, suggesting that parents with higher education may have greater access to lactation information and support, and may therefore be more persistent in maintaining milk supply (26). The increased physiological and logistical demands on mothers of multiples may necessitate more targeted clinical interventions.

Interestingly, in our study, lower gestational age was found to be significantly associated with higher EB rates at 6 months. Although higher gestational age is often associated with greater success, this finding mirrors results from other European and Israeli studies (6, 9, 13, 26). One possible explanation is that mothers of more vulnerable infants may be more motivated to breastfeed, and healthcare staff may provide more intensive support for these infants due to their high-risk situation. Additionally, in our study, EB groups were found to have longer times to achieve first total enteral or total oral feedings, which could also be explained by the tendency to prioritize breastfeeding in high-risk infants.

In our study, an association was found between a younger age at formula initiation and no breastfeeding status at 6 and 12 months. Infants who were still being breastfed at 12 months had initiated formula significantly later—an average of 3.6 months later—than those who had ceased (*p* = 0.007). This finding reinforces the well-known negative association between formula introduction and breastfeeding continuation (27, 28).

Our study presents exclusive and total breastfeeding durations calculated according to both chronological and corrected ages. A study from China reported an EB duration of 1.9 ± 3.3 months after discharge in very preterm infants (9). Similarly, a Portugese study reported a median EB duration of 2 months chronological age and a median total breastfeeding duration of 3 months chronological age in very preterm infants (12). The public report from our country indicated a median total breastfeeding duration of 16.7 months (15). Breastfeeding duration of our group is longer than those reported internationally but shorter than that of term infants, and all fall considerably short of WHO recommendations. These findings underscore that even in countries with high initiation rates, very preterm infants remain at risk for early weaning.

An additional issue identified was the misinterpretation of chronological versus corrected age for feeding decisions. Approximately one-thirds of infants were introduced to complementary foods at 6 months of chronological age, not corrected age, which may have played a role in shorter exclusive and total breastfeeding durations. Continuous guidance during follow-up visits emphasizing corrected age may help prevent early weaning.

Prior research has shown that partial breastfeeding at discharge is associated with earlier cessation of breastfeeding (13, 29, 30). Our findings strongly support this association with high effect sizes; exclusive breastfeeding at discharge emerged as the most strongly associated factor for long-term success, increasing the likelihood of breastfeeding at 12 months by nearly eight-fold. This predictive value remained significant also at 18 months. This underscores the need for NICU policies aimed at achieving higher EB rates before discharge as a one of the primary clinical levers for long-term breastfeeding continuity.

Studies from various countries report early introduction of complementary feeding in preterm infants. A US study found that 64.5% of preterm infants were introduced before 4 months of age, and Italian and Australian studies reported averages of 14–15 weeks (31–33). In our study, although the timing was closer to recommendations, half of the infants began before 6 months, suggesting the need for more tailored counseling.

Mothers' satisfaction with breastfeeding duration was also suboptimal: 61.1% reported that they had not breastfed as long as they wished. This aligns with prior findings from Sweden, where 43% of mothers expressed similar dissatisfaction (11). These findings support the idea that preterm infants' mothers will react positively to increasing lactation support at NICU and follow-up care.

It is important to clarify the scope of this study within the national context. While our data are derived from a single tertiary center, our unit operates under the strict “Baby-Friendly NICU” protocols mandated by the Turkish Ministry of Health. Therefore, although this is not a nationally representative epidemiological survey, it provides the first longitudinal reflection of long-term breastfeeding outcomes for very preterm infants within the framework of Türkiye's national healthcare policies.

## Limitations

The main limitations of this study are the relatively small sample size, retrospective design, and partial reliance on maternal recall. However, prior research indicates that mothers' recall of breastfeeding information remains reliable for several years (34, 35). Efforts were made to minimize recall bias by including only infants born within the previous three years and verifying information through interviews.

Additionally, due to the relatively small sample size, multivariate analysis was not performed to identify independent predictors of breastfeeding outcomes. This constraint limits the ability to control for all potential confounding factors, such as maternal stress levels or specific NICU clinical interventions, which may have influenced breastfeeding duration. Future larger-scale prospective studies are needed to conduct such analyses and provide a more comprehensive understanding of these independent factors.

## Conclusions

Our study demonstrated relatively low breastfeeding rates and short durations among very preterm infants, emphasizing the need for targeted clinical levers. In particular, reinforcing exclusive breastfeeding practices within the NICU to achieve higher rates at discharge may serve as a critical actionable factor for long-term success. Enhancing lactation support during follow-ups—with a specific focus on mitigating unnecessary formula introduction and addressing sociodemographic factors such as education levels and plurality—may play a key role in improving outcomes. By implementing these targeted interventions, such as intensive predischarge counseling and structured outpatient follow-ups, and providing comprehensive support to mother-infant dyads, healthcare professionals can bridge the gap between hospital success and sustained long-term breastfeeding.

## Data Availability

The raw data supporting the conclusions of this article will be made available by the authors, without undue reservation.
